# Porous Calcium Phosphate Ceramic Scaffolds with Tailored Pore Orientations and Mechanical Properties Using Lithography-Based Ceramic 3D Printing Technique

**DOI:** 10.3390/ma11091711

**Published:** 2018-09-13

**Authors:** Jung-Bin Lee, Woo-Youl Maeng, Young-Hag Koh, Hyoun-Ee Kim

**Affiliations:** 1 School of Biomedical Engineering, Korea University, Seoul 02841, Korea; jungbin15@nate.com (J.-B.L.); abcd2165@korea.ac.kr (W.-Y.M.); 2 Department of Materials Science and Engineering, Seoul National University, Seoul 08826, Korea; kimhe@snu.ac.kr

**Keywords:** additive manufacturing, 3D printing, photocuring, porous ceramics

## Abstract

This study demonstrates the usefulness of the lithography-based ceramic 3-dimensional printing technique with a specifically designed top-down process for the production of porous calcium phosphate (CaP) ceramic scaffolds with tailored pore orientations and mechanical properties. The processing parameters including the preparation of a photocurable CaP slurry with a high solid loading (*φ* = 45 vol%), the exposure time for photocuring process, and the initial designs of the porous scaffolds were carefully controlled. Three types of porous CaP scaffolds with different pore orientations (i.e., 0°/90°, 0°/45°/90°/135°, and 0°/30°/60°/90°/120°/150°) were produced. All the scaffolds exhibited a tightly controlled porous structure with straight CaP frameworks arranged in a periodic pattern while the porosity was kept constant. The porous CaP scaffold with a pore orientation of 0°/90° demonstrated the highest compressive strength and modulus due to a number of CaP frameworks parallel to the loading direction. On the other hand, scaffolds with multiple pore orientations may exhibit more isotropic mechanical properties regardless of the loading directions. The porous CaP scaffolds exhibited an excellent in vitro apatite-forming ability in a stimulated body fluid (SBF) solution. These findings suggest that porous CaP scaffolds with tailored pore orientations may provide tunable mechanical properties with good bone regeneration ability.

## 1. Introduction

Calcium phosphate (CaP) ceramics, which have chemical compositions and crystalline structures similar to those of the inorganic phase of natural bones [1], are biocompatible, biodegradable, and osteo-conductive in vivo [2]. Thus, they can form direct physicochemical bonds with surrounding bone when used as bone substitutes in dental and orthopedic applications [3,4]. In addition, when formulated into the porous structures, porous CaP ceramic scaffolds can accelerate bone regeneration by stimulating bone ingrowth into pores [5,6]. However, as the porosity increases, the mechanical strength of these scaffolds decreases dramatically, which limits the range of potential clinical applications (e.g., load-bearing bone scaffolds) [1,5,6].

To overcome this limitation, 3D printing and additive manufacturing (AM) techniques have recently gained significant attention in the production of porous ceramic and metallic scaffolds since they can provide high mechanical properties without sacrificing high porosity required for fast bone regeneration [7,8,9,10,11,12,13,14]. These techniques can create arbitrarily-designed porous ceramic scaffolds with external and internal structures by selectively consolidating ceramic-based feed stocks in a layer-by-layer fashion, according to predetermined 3D models [15,16,17]. The ability of these AM techniques to construct high-quality architectures with complex shapes is strongly affected by the consolidation mechanism of the ceramic-based feed stocks and the tool associated with the process.

Extrusion-based AM techniques can create three-dimensionally interconnected pore networks by depositing green ceramic filaments extruded through fine nozzles, according to predetermined printing paths [18,19,20,21,22,23,24,25,26]. In addition, they can readily tune the porosity and pore size of porous ceramic scaffolds simply by adjusting the distance between the deposited filaments, which provides tailored mechanical properties. However, ceramic networks generally have simple geometries (e.g., circular or rectangular cross-sections) due to the limited range of nozzle designs for extrusion. Furthermore, these techniques have a limited ability to construct the arbitrarily designed external shapes and internal pore architectures required for individual patients since they generally require a continuous deposition process without supporting layers.

By comparison, photo-polymerization-based AM techniques (e.g., stereolithography (SLA) [27] and digital light processing (DLP) [28]) can create more complex shapes with high accuracy and smooth surfaces since they can selectively and precisely polymerize thin layers of photo-curable ceramic slurries using high-resolution light engines [29,30]. Bioactive ceramics such as bioactive glasses [28,31,32,33,34], glass-ceramics [35], and CaP ceramics [36,37] have been investigated as the scaffold materials with the aim to construct extremal shapes and internal porous structures that are tailored for individual patients. However, to achieve sufficiently high quality, several processing parameters need to be closely controlled including the preparation of the photo-curable ceramic slurries and control over their curing behavior as well as the heat-treatment of green scaffolds for de-binding and sintering at high temperatures [28,30,32]. More specifically, ceramic slurries require a high solid loading for high densification after sintering while, at the same time, preserving the low viscosity and high flowability required for the creation of thin layers for a layer-by-layer photocuring process. In addition, the curing conditions such as the energy dose and exposure time should be optimized to ensure the effective polymerization of ceramic layers while minimizing the line broadening due to ultraviolet (UV) scattering by ceramic particles. The de-binding and sintering process should also be carefully designed to avoid the formation of defects such as distortions, large pores, and cracks. These complicated factors limit the wider use of lithography-based AM techniques in the production of porous ceramic scaffolds with advanced functions.

In this study, we demonstrate the utility of lithography-based ceramic AM technique for the production of porous CaP scaffolds with tailored pore orientations and mechanical properties. Hexanediol diacrylate (HDDA) and decahydronaphthalene (decalin) were used as the photo-curable monomer and diluent, respectively, in order to achieve a high solid loading with the desired rheological behavior. In addition, unlike other lithography-based AM techniques, a fresh new layer of the CaP slurry was directly deposited onto the previously photo-cured layer using a custom-made recoating device. Line broadening caused by the UV scattering effect due to CaP ceramic particles was carefully examined in order to precisely control the porous structure of the CaP scaffolds. In order to tailor mechanical properties, three types of porous CaP scaffolds with different pore orientations (i.e., 0°/90°, 0°/45°/90°/135°, and 0°/60°/90°) were produced.

The porous structure (e.g., porosity, pore size, and pore geometry) of the porous CaP scaffolds and the microstructure of the CaP frameworks were characterized by optical microscopy and field emission scanning electron microscopy (FE-SEM). The crystalline structure and phases were examined by X-ray diffraction (XRD, M18XHF-SRA, MacScience Co., Yokohama, Japan). The mechanical properties were characterized using compressive strength tests. To evaluate the potential of the porous CaP scaffolds as the bone scaffold, their in vitro apatite-forming ability was examined.

## 2. Materials and Methods

### 2.1. Starting Materials

As the scaffold material, commercially available calcium phosphate powder (CaP, OssGen Co., Daegu, Korea), comprised of hydroxyapatite (HA) and β-tricalcium phosphate (β-TCP) with a weight ratio of 60:40 was used. The particle size of the as-received CaP powder was measured using a laser diffraction particle size analyzer (Cilas 1090, Orleans, France). Polypropylene oxide quaternary ammonium chloride (VARIQUAT® CC-9, Evonik, Germany) was used as the dispersant. The 1,6-Hexanediol diacrylate (HDDA, Sartomer, Exton, PA, USA), decahydronaphthalene (Decalin, Sigma Aldrich, St. Louis, MO, USA), Phenylbis (2,4,6-trimethyl benzoyl)phosphine oxide (PPO, Sigma Aldrich, St. Louis, MO, USA) were used as the photocurable monomer, the diluent, and the photo initiator, respectively.

### 2.2. Photocurable Ceramic Slurry Preparation

The photo curable formulation was prepared by mixing HDDA and decalin by magnetic stirring. A predetermined amount of CaP powder was then added into the HDDA/decalin mixture with the assistance of 4 wt% of dispersant (Table 1). Various solid loadings (*φ* = 10 vol%, 30 vol%, 40 vol%, and 45 vol%) were used to examine the optimized rheological behavior of the CaP slurry for 3D printing purposes. The individual CaP slurries were then vigorously mixed using a paste mixer (Hantech Co, Ltd., Hwaseong-si, Korea) for 1 h at 1000 rpm, which was followed by ball-milling for 24 h. Before the 3D printing process, 2 wt% of the photo initiator was added to the prepared CaP slurries and then mixed for 0.5 h using the paste mixer.

### 2.3. Rheological Behavior Analysis

The viscosity of the CaP slurries with various CaP contents (*φ* = 10 vol%, 30 vol%, 40 vol%, and 45 vol%) was measured using a viscometer (DV-E Viscometer, Bookfield, MA, USA). The rheological behavior of the most highly concentrated CaP slurry (*φ* = 45 vol%) was more closely characterized by using a cone/plate viscometer (DV3T-CP, Bookfield, MA, USA). The apparent viscosity of the CaP slurry was monitored as a function of the shear rate ranging from 0.1 s^−1^ to 100 s^−1^.

### 2.4. Photocuring Behavior Analysis

The photo curing behavior of the most highly concentrated CaP slurry was characterized by using a differential scanning calorimeter (DSC, Q 1000, TA Instruments, New Castle, DE, USA), which can quantitatively assess reactions and kinetics during photo polymerization [38,39]. During the DSC measurement process, the heat flow evolved from the sample and was monitored as a function of time. The percent conversion (*α*_t_) was calculated by considering the total heat evolved at time *t*, Δ*H*(*t*), which is shown below.

*α*_t_ = [Δ*H*(*t*)/n·(Δ*H*_0_·m)] (1)

where n is the number of C=C bonds per monomer (n = 2 for HDDA), Δ*H*_0_ is the standard enthalpy of reaction for the acrylate double bond (Δ*H*_0_ = 86.2 kJ mol^−1^ for HDDA), and m is the moles of acrylate monomer in the sample [38].

### 2.5. Custom-Built 3D Printing Machine Set-Up

To produce porous CaP scaffolds with tailored pore orientations, a custom-built 3D printing machine was set up. A DLP with a digital micro-mirror device (DMD) employing a dynamic mask of 1920 × 1080 pixels (HD25-LV, Optoma, Fremont, CA, USA) was used as the light engine, which emits UV light at a power of ~16 mW and a peak wavelength of ~405 nm. Unlike conventional DLP processes, our approach can fabricate 3D objects through top-down processing, which means a thin layer of the ceramic slurry can be deposited directly onto a previously photo cured layer.

### 2.6. Cure Depth, Cure Width, and Line Broadening Analysis

The layers of the CaP slurry were photo cured for various times (5 s, 7 s, 10 s, and 17 s) using our custom-built 3D printer. Subsequently, the thickness of the photo cured layers was measured using a micrometer. In addition, line broadening due to UV scattering by ceramic particles was examined. Long strips with widths ranging from 90 μm to 900 μm were printed onto a 200-μm thick layer for 7 s. The widths of the photo cured strips were calculated from the SEM images and compared to those of the initial designs.

### 2.7. Porous CaP Scaffolds Fabrication

**Figure 1:**
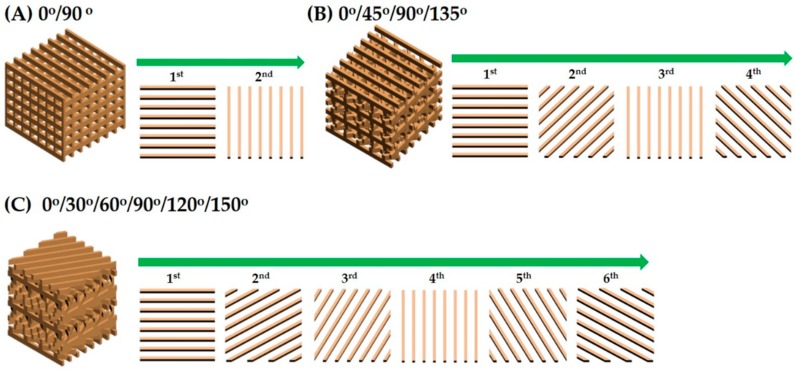
Schematic diagram showing the porous structures of various CaP scaffolds with different pore orientations: (**A**) 0°/90°, (**B**) 0°/45°/90°/135°, and (**C**) 0°/30°/60°/90°/120°/150° with their top-down view of the repeating CaP frameworks.

Three different types of porous CaP scaffolds that differed in their pore orientations (i.e., 0°/90°, 0°/45°/90°/135°, and 0°/30°/60°/90°/120°/150°) were designed as a way to control mechanical properties of the scaffolds (Figure 1). Each scaffold was composed of straight CaP frameworks arranged in a controlled, periodic pattern.

The dimensions of the CaP frameworks were 450 μm × 1 mm in the x–z direction for all scaffolds while those of the channels were 1.5 mm × 1 mm (Table 1). As a result, the three scaffolds had similar porosities (~75 vol%) but with different pore orientations. In the layer-by-layer printing process, 200-μm thick layers of the CaP slurry were used to construct CaP frameworks. Each layer was photo cured for 7 s. Five layers were used to construct the 1 mm-thick CaP frameworks.

### 2.8. Debinding and Sintering Process

The weight loss of the sample was monitored by TGA analysis (thermogravimetric analysis) (DTG-60, Shimadzu Co., Ltd. Kyoto, Japan). The sample was heat-treated up to 600 °C at a heating rate of 10 °C/min in the air and its weight loss was recorded. Based on this TGA analysis, a de-binding schedule was established and this is summarized in Table 2. After de-binding, the porous CaP scaffolds were sintered at 1250 °C for 3 h to densify the CaP filaments.

### 2.9. Porous Structure Evaluation

The porous structure and microstructure of the as-built and sintered CaP scaffolds were examined by optical microscopy and field emission scanning electron microcopy (FE-SEM, JSM-6701F, JEOL Techniques, Tokyo, Japan). The dimensions of the CaP frameworks and pores in the x–z direction were measured from the SEM images. The overall porosity (*P*) of the porous CaP scaffolds was calculated by dividing its apparent density (*ρ*_a_) by the theoretical density (*ρ*_s_) of the dense CaP ceramic, which is shown below.

*P* (%) = 100 − 100·(*ρ*_a_/*ρ*_s_) (2)

### 2.10. Compressive Strength Testing

The mechanical properties of three porous CaP scaffolds with different pore orientations (i.e., 0°/90°, 0°/45°/90°/135°, and 0°/30°/60°/90°/120°/150°) were evaluated using compressive strength tests. Specimens with dimensions of ~11 × 11 × 11 mm were uni-axially compressed at a crosshead speed of 1 mm/min using a screw-driven load frame (OTU-05D; Oriental TM Corp., Gyeonggi-do, Korea). A compression load was applied to the side of the scaffold, which means the CaP frameworks with an orientation of 0° were parallel to the loading direction. During testing, the stress and strain responses of the specimens were monitored. Five specimens for each scaffold type were tested to obtain the mean value and standard deviation.

### 2.11. In vitro Apatite-Forming Ability Evaluation

The in vitro apatite-forming ability of the porous CaP scaffold was characterized using a stimulated body fluid (SBF) solution, which was prepared according to the method reported in the literature [40,41]. The formation of apatite layers on the porous CaP scaffolds after 3 days of immersion in the SBF solution was examined by FE-SEM.

## 3. Results and Discussion

### 3.1. Characteristics of Starting CaP Powder

**Figure 2:**
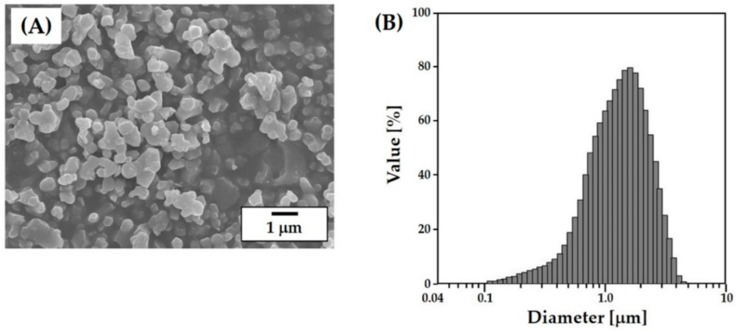
(**A**) Representative SEM image of the as-received CaP powders and (**B**) the particle size distribution of the as-received CaP powders.

One of the most crucial steps in the lithography-based ceramic 3D printing process is to prepare proper ceramic slurries that have a high solid loading for high densification after sintering and a reasonably low viscosity for the creation of thin layers during layer-by-layer printing [25,28]. The characteristics of the starting ceramic powder used in the process including particle shape, particle size and its distribution, and surface area strongly affect the rheological behavior of a highly concentrated ceramic slurry. The as-received CaP powder exhibited a well-defined morphology without severe agglomerations, which is shown in Figure 2A. The particle sizes, which are measured using a laser diffraction particle size analyzer, ranged between ~0.6 and 2.62 μm. The mean particle size was 1.38 μm (Figure 2B). This powder characteristic is conducive to the preparation of highly concentrated CaP slurries given that the fine CaP powders can be uniformly dispersed in photo curable monomers while avoiding the severe sedimentation of the CaP powders in the slurry during the 3D printing process.

### 3.2. Rheological Behavior of CaP Slurries

**Figure 3:**
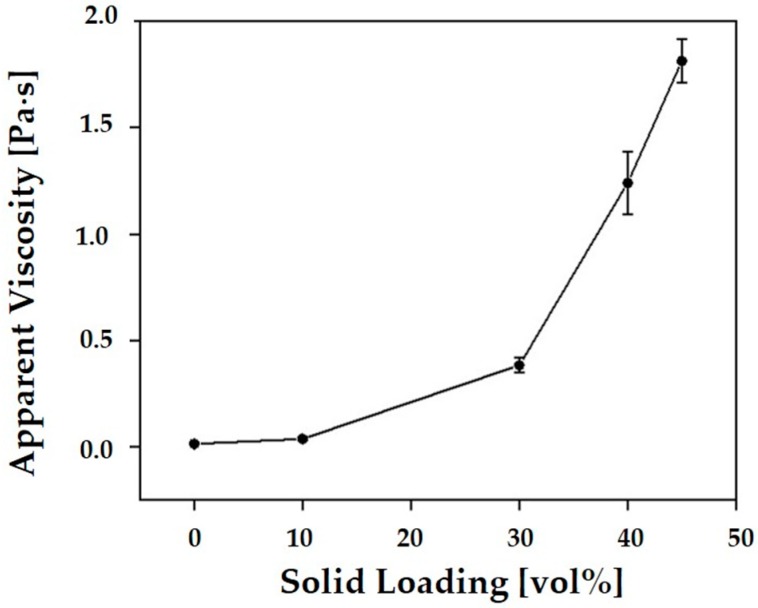
Apparent viscosity of the CaP slurries as a function of solid loading (*φ* = 10 vol%, 30 vol%, 40 vol%, and 45 vol%).

The effect of solid loading on the viscosity of the CaP slurries was examined using a viscometer, which is shown in Figure 3. The viscosity of the slurries increased with an increase in solid loading. This increase was particularly large for solid loadings higher than 40 vol%. However, it should be noted that the most highest solid loading (*φ* = 45 vol%) still had a reasonably low viscosity of ~1.74 Pa·s, which means it could be successfully used as the feedstock for the proposed lithography-based ceramic 3D printing process [33].

**Figure 4:**
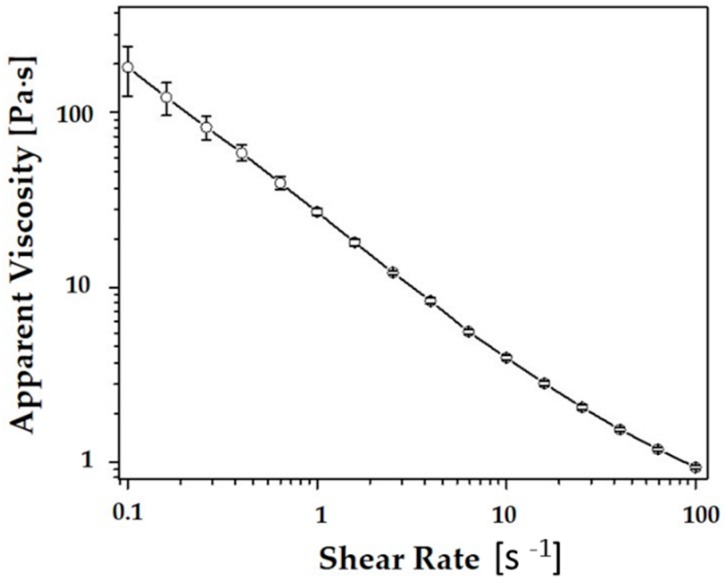
Apparent viscosity of the CaP slurry as a function of shear rate (*φ* = 45 vol%).

The rheological behavior of the most highly concentrated CaP slurry (*φ* = 45 vol%) was more closely examined using a cone/plate rheometer. The apparent viscosity of the slurry decreased almost linearly with an increase in the shear rate, which is shown in Figure 4. This shear thinning behavior is highly beneficial for the lithography-based ceramic 3D printing process. More specifically, CaP ceramic slurries should have a reasonably low viscosity (e.g., <20 Pa·s) at relatively high shear rates [33] so that thin, uniform layers can be deposited onto the building platforms using a recoating system. In addition, they are preferred to have a reasonably high viscosity at rest to maintain the uniform structure of the layers during the printing process. The measured viscosity at the shear rates of ~100 s^−1^ and ~0.1 s^−1^ were ~1.24 Pa·s and ~286 Pa·s, respectively. This finding suggests that the CaP slurry prepared in this study can be effectively used as the feedstock for our 3D printing system.

### 3.3. Photocuring Behavior of the CaP Slurry

**Figure 5:**
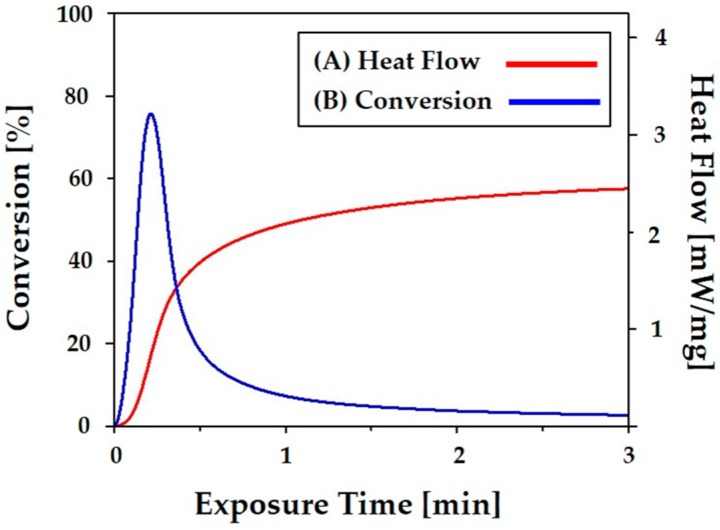
Photo differential scanning calorimetry (photo-DSC) results for the highly concentrated CaP slurry showing (**A**) heat flow and (**B**) % conversion as a function of exposure time.

The photo curing behavior of the most highly concentrated CaP slurry was examined using photo calorimetry, which can quantify the reactions and kinetics of photo polymerization [33]. Figure 5A displays the heat flow that evolved from the sample during photo curing for up to 5 min. A single exothermic curve was observed due to the free-radical photo polymerization reaction of the HDDA monomer [38]. The heat flow increased very rapidly with an increase in exposure time up to ~12 s, which was followed by a rapid drop. This photo curing behavior is attributed to the auto acceleration and autocatalytic reaction of the bulk polymerization reaction, which is commonly occurs in polymerization reactions. The polymerization reaction terminated within ~3 min, which would correspond to ~12 mJ·cm^−2^ at a constant intensity of 20 mW·cm^−2^.

In order to more closely examine the photo curing behavior of the CaP slurry, the percent conversion (*α*) of the CaP slurry was calculated based on the exothermic heat flow at time *t* and the expected heat of polymerization of the HDDA monomer, which is shown in Figure 5B. The percentage conversion increased very rapidly with an increase in exposure time up to ~12 s and then continued to increase up to ~3 min. The final conversion was ~88%. This value would provide a reasonably high green strength for the construction of porous CaP scaffolds. This finding suggests that the photo curing of the most highly concentrated CaP slurry can occur very rapidly, which would facilitate a rapid building speed. It should be noted that the photo curing behavior the CaP slurry can be further tailored by adjusting the photo initiator if required.

### 3.4. Control over Cure Depth, Cure Width, and Line Broadening

**Figure 6:**
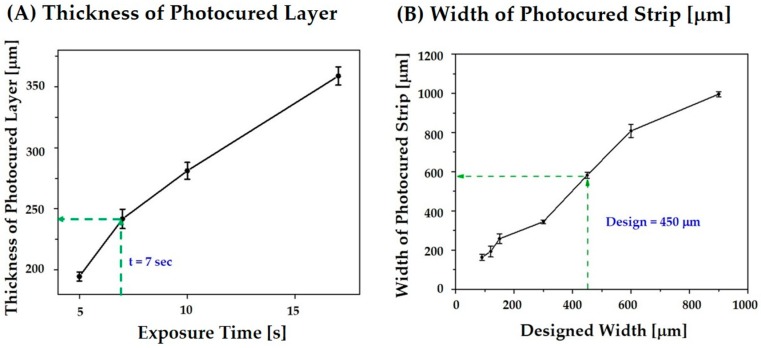
(**A**) Thicknesses of the photo cured layers as a function of exposure time at a constant UV power of 16 mW and (**B**) measured widths of the photo cured strips as a function of their designed widths after photo curing for 7 s.

Exposure time, which determined the UV energy dose for the photo curing of the highly concentrated CaP slurry, was assessed to ensure strong bonding between the photo cured layers while minimizing the over curing phenomenon. The CaP slurry was photo cured for various times (5 s, 7 s, 10 s, and 17 s) at a constant UV power of ~16 mW. The thickness of the photo cured layers, which was measured using a micrometer, increased from 192 μm to 356 μm with an increase in exposure time from 5 to 17 s, which is shown in Figure 6A. This finding suggests that an exposure time of 7 s would effectively photo cure the 200-μm layers of the CaP slurry used as the feedstock for the production of porous CaP scaffolds while preserving the open porous structures by minimizing the over curing phenomenon.

To tightly control the porous structure of the CaP scaffolds (e.g., the dimensions of the CaP frameworks and channels), the line broadening due to UV scattering by CaP powders was carefully examined. Long strips with various widths ranging from 90 μm to 900 μm were photo cured for 7 s, which is an exposure time chosen on the basis of the previous cure depth measurements. The widths of the photo cured strips were calculated from the SEM images and compared to those of the initial designs, which is shown in Figure 6B. Significant increases in width were observed for all of the designs. The results of UV scattering by the CaP powders is in the slurry [30]. The degree of line broadening increased linearly with an increase in the initial width. In other words, a thicker design would result in a much thicker strip [32]. This finding suggests that the width of the CaP frameworks can be specifically controlled by adjusting the initial designs. For example, an initial width of 450 μm can be used to produce ~600-μm-thick CaP frameworks.

### 3.5. Thermal Behavior of Photocured CaP

**Figure 7:**
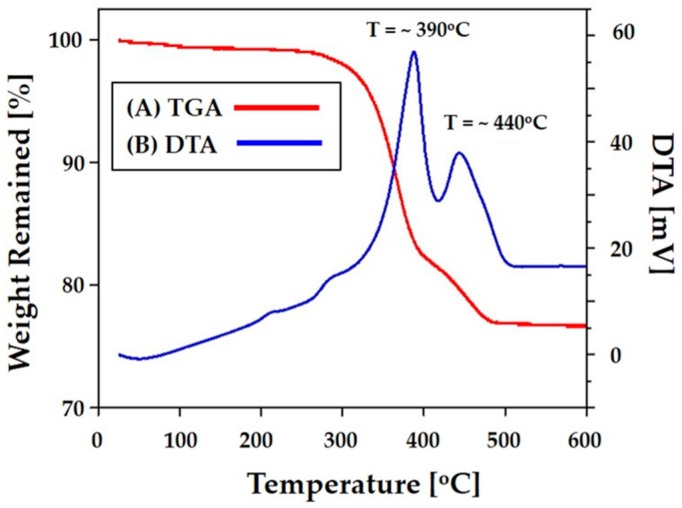
(**A**) TGA and (**B**) DTA results for the photo cured CaP sample as a function of temperature.

The thermal behavior of the photo cured CaP sample was characterized using a TGA-DTA (Differential thermal analysis) analysis for the purpose of optimizing the de-binding process. Figure 7A,B present the weight loss and temperature difference of the photo cured CaP sample, respectively, as a function of temperature. Weight loss, which was attributed to the thermal decomposition of the photo cured polymer and additives (e.g., the diluent, dispersant, and photo initiator), was observed at temperatures in the range of ~212 °C to ~500 °C (Figure 7A). In addition, extensive exothermic reactions occurred at ~390 °C and 440 °C (Figure 7B). Thus, special care was taken to remove the polymers slowly within these temperature ranges (c.f. Table 2) to avoid the formation of defects. It should be noted that the final content of the CaP in the photo cured CaP sample was ~75.7 wt%, which corresponded to a volume of ~52 vol%. This is higher than the initial content in the CaP slurry. The difference is presumably due to the removal of decalin, which was used as a diluent, during the 3D printing process. To densify the CaP frameworks, the as-built CaP scaffolds were finally sintered at 1250 °C for 3 h.

### 3.6. Porous Structure and Microstructure of As-Built Porous CaP Scaffolds

**Figure 8:**
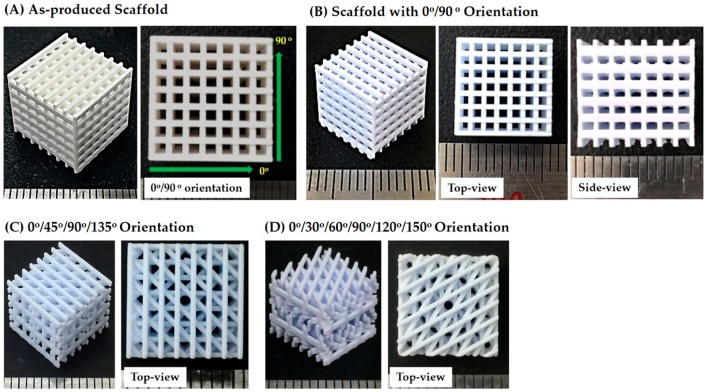
Optical images of (**A**) the as-built CaP scaffold showing its overall porous structure and the construction of straight CaP frameworks in in a 0°/90° orientation and the porous CaP scaffolds after sintering at 1250 °C for 3 hours, which have different pore orientations of (**B**) 0°/90°, (**C**) 0°/45°/90°/135°, and (**D**) 0°/30°/60°/90°/120°/150° (scale = 1 mm).

Porous CaP scaffolds with a pore orientation of 0°/90° were produced using predetermined processing parameters (thickness of the individual CaP slurry layers = 200 μm, exposure time = 7 s, and initial width of the CaP framework = 450 μm). The overall porous structure of the as-built CaP scaffolds was characterized by using optical microcopy, which is shown in Figure 8A. The scaffold exhibited a tightly controlled 3D porous structure without noticeable defects such as distortion and the clogging of channels caused by a residual slurry. In addition, straight CaP frameworks of similar widths were uniformly created throughout the scaffold in a 0°/90° orientation.

The dimensions of the CaP frameworks and channels were ~583 (±12) μm × 1059 (±18) μm and ~1194 (±17) μm × 1059 (±18) μm in the x–z direction, respectively (c.f. Table 1). The overall porosity of the as-built scaffolds was 80 (±18) vol%, which was slightly larger than that of the initial design (75 vol%).

**Figure 9:**
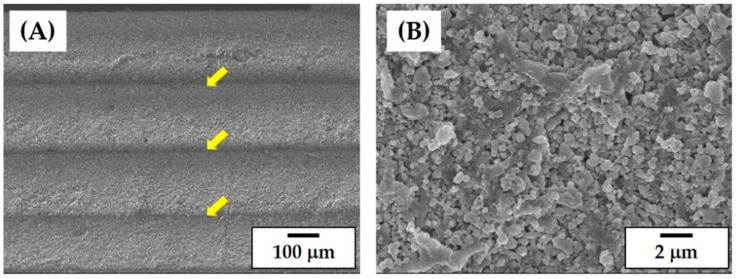
Representative SEM images of the CaP framework showing (**A**) its surface morphology in the building direction and (**B**) its microstructure.

The surface morphology and microstructure of the CaP frameworks were more closely examined by FE-SEM, which is shown in Figure 9A,B. In the building direction, the layers used for the construction of the CaP framework are visible. However, they were strongly bonded together, which is marked by the yellow arrows (Figure 9A). The photo cured layers were slightly distorted, which is presumably due to the parabolic curing pattern of the CaP slurry by UV light [32]. However, this distortion would be mitigated by using thinner layers of the CaP slurry for the layer-by-layer printing process if necessary. The microstructure of the photo cured CaP framework is shown in Figure 9B. The CaP powders were uniformly distributed throughout the CaP framework.

### 3.7. Porous Structure of Porous CaP Scaffolds

The porous CaP scaffolds with different pore orientations (i.e., 0°/90°, 0°/45°/90°/135°, and 0°/30°/60°/90°/120°/150°) were obtained after sintering at 1250 °C for 3 h, which is shown in Figure 8B–D. All of the produced scaffolds tightly controlled pore configurations. Straight CaP frameworks in controlled patterns were constructed throughout the scaffolds. A linear shrinkage of ~21% was observed after sintering at 1250 °C for 3 h. The dimensions of the CaP frameworks and channels were kept consistent and, thus, similar porosities (~70 vol%) were obtained for all of the scaffolds (Table 1). These values are lower than that of the as-built scaffold, which would be due to the considerable shrinkage of the CaP frameworks. The dimensions of the CaP frameworks were ~484 (±8) μm × 583 (±12) μm in the x–z direction while those of the channels were ~951 (±9) μm × 1194 (±18) μm. These finding suggests that the porous structures of the porous CaP scaffolds (e.g., porosity and pore size) can be readily tailored by adjusting the initial 3D designs.

### 3.8. Microstructure of Porous CaP Scaffolds

**Figure 10:**
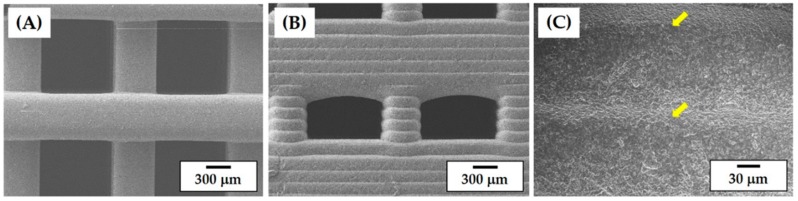
Representative FE-SEM images of the porous CaP scaffolds showing the surface morphologies of (**A**) the top-view and side-views (**B**) at low and (**C**) at high magnifications.

The porous structure and microstructure of the CaP scaffolds with a pore orientation of 0°/90° were more closely characterized by FE-SEM, which is shown in Figure 10A–C. A tightly controlled 3D porous structure with three-dimensionally interconnected channels was achieved for the scaffolds with a pore orientation of 0°/90° (Figure 10A). No noticeable defects (e.g., distortion or cracking) were observed due to the use of the specially designed heat-treatment for de-binding and sintering. The straight CaP frameworks were uniformly constructed at a 0°/90° orientation throughout the scaffold. The sintered CaP frameworks exhibited relatively smooth surfaces without noticeable features (Figure 10A) while the five layers composed of the CaP frameworks are clearly visible in the building direction (i.e., z direction), which resembles the structure of the as-built scaffold (Figure 10B). However, they were strongly bonded together without delamination, which was marked by the yellow arrows (Figure 10C). These surface morphologies are useful for the attachment, proliferation, and differentiation of osteoblasts when used as bone scaffolds [38,39].

**Figure 11:**
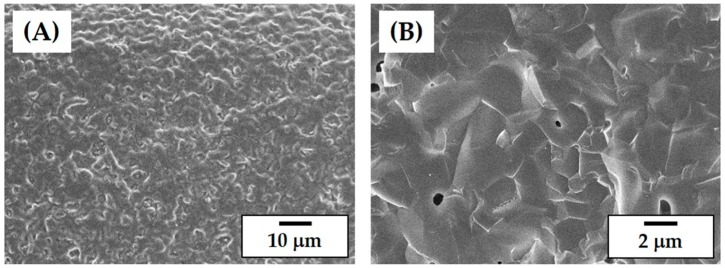
Representative FE-SEM images of (**A**) the free surface and (**B**) fracture surface of the CaP frameworks.

The free and fracture surfaces of the CaP frameworks are presented in Figure 11A,B, respectively. The CaP frameworks revealed high densification but a rough surface (Figure 11A), which is advantageous for the attachment, proliferation, and differentiation of osteoblasts when used as bone scaffolds [42,43]. In addition, only some residual pores were observed on the fracture surface (Figure 11B). This promising densification behavior is due to the high solid loading (45 vol%) and the carefully designed heat-treatment for de-binding and sintering.

### 3.9. Control of Mechanical Properties

**Figure 12:**
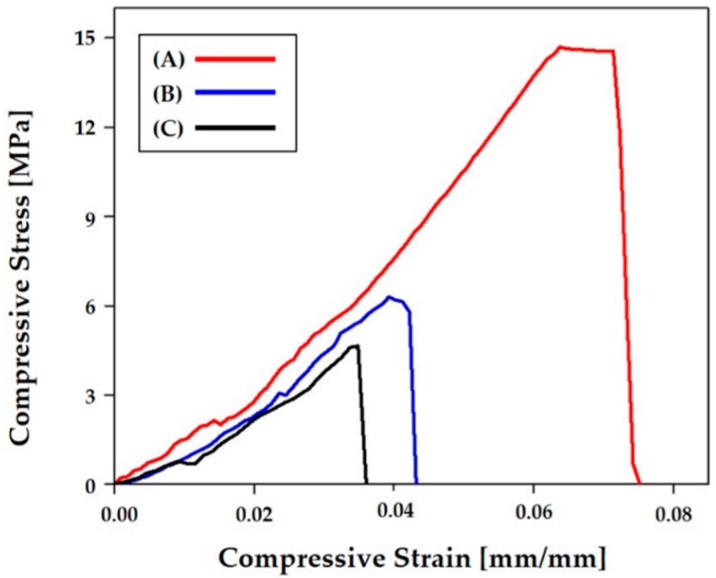
Representative compressive stress versus strain responses of porous CaP scaffolds with different pore orientations of (**A**) 0°/90°, (**B**) 0°/45°/90°/135°, and (**C**) 0°/30°/60°/90°/120°/150°.

The effect of pore orientation on the mechanical properties of the porous CaP scaffolds made up of straight CaP frameworks was examined using compressive strength tests. Porous CaP scaffolds with three different pore orientations (i.e., 0°/90°, 0°/45°/90°/135°, and 0°/30°/60°/90°/120°/150°) were tested with compressive loads applied normally to the printing direction (in the z direction, insets in Figure 12A–C). Regardless of the pore orientations, all three porous CaP scaffolds exhibited similar fracture behaviors with the compressive stress increasing linearly and then dropping very rapidly. This mechanical response can be attributed to the brittle nature of the CaP frameworks, which is often the case with porous ceramics [44]. However, it should be noted that the compressive stress and strain at the fracture, which are marked by the arrows, were strongly affected by the pore orientation.

The compressive strength and modulus of the porous CaP scaffolds with different pore orientations were computed from their compressive stress versus strain responses, which is summarized in Table 3. The porous CaP scaffold with a pore orientation of 0°/90° had the highest compressive strength (14.9 ± 1.61 MPa) and compressive modulus (274 ± 24.4 MPa). These values are comparable to those of porous CaP scaffolds with similar porous structures consisting of straight CaP frameworks arranged in a periodic pattern [23,37,45].

**Figure 13:**
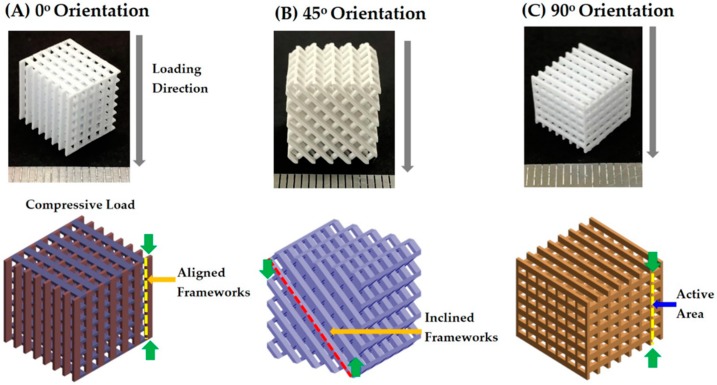
Optical images of the porous CaP scaffolds with a pore orientation of 0°/90°, which were compressed at the loading directions of (**A**) 0°, (**B**) 45°, and (**C**) 90° (building direction). Schematic diagrams represent the frameworks placed under compressive or bending stress.

Mechanical properties such as the compressive strength and modulus of the porous CaP scaffolds can be tailored by controlling their pore orientation. More specifically, the CaP frameworks with an orientation of 0°, which are parallel to the loading direction, can more effectively withstand applied compressive loads (Figure 13A). Thus, the porous CaP scaffold with a pore orientation of 0°/90° can have the highest compressive strength (14.9 ± 1.61 MPa). However, when this scaffold was subjected to an inclined compressive load at θ = 45° (Figure 13B), much lower compressive strength (0.73 ± 0.1 MPa) was obtained since the CaP frameworks were likely to be fractured by bending and buckling stresses [45,46,47]. This value was much lower than those of the porous CaP scaffolds with pore orientations of 0°/45°/90°/135° (6.2 ± 1.10 MPa) and 0°/30°/60°/90°/120°/150° (4.8 ± 0.35 MPa). Thus, it is reasonable to suppose that the porous CaP scaffolds with multiple pore orientations are expected to exhibit more isotropic mechanical properties since several frameworks can be parallel to the loading direction regardless of the loading directions (c.f. Figure 1). In addition, when compressed in the building direction (Figure 13C), the porous CaP scaffold with a pore orientation of 0°/90° showed a reasonably high compressive strength of 6.7 ± 1.34 MPa since the active areas can effectively withstand applied compressive loads. This finding suggests that the lithography-based ceramic 3D printing process used in this study can readily tailor and/or optimize the pore orientation of porous CaP scaffolds to provide advantageous mechanical properties with an excellent bone regeneration ability [46].

### 3.10. In Vitro Apatite-Forming Ability

**Figure 14:**
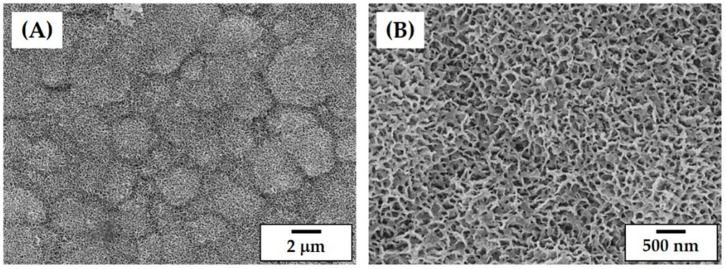
Representative FE-SEM images of the surface morphologies of the CaP frameworks showing the deposition of apatite crystals after immersion in an SBF solution for 1 day (**A**) at low and (**B**) at high magnifications.

In order to evaluate the potential of the porous CaP scaffold as a bone scaffold, its in vitro apatite-forming ability was examined using an SBF solution. Figure 14A,B present representative SEM images of the surface morphologies of the CaP frameworks after the immersion in the SBF solution for 1 day. The surface of the CaP frameworks was entirely covered with the clusters of apatite crystals (Figure 14A). In addition, these clusters consisted of a number of tiny apatite nanocrystals (Figure 14B), which is often the case with bioactive CaP ceramics [3,4]. This finding suggests that porous CaP ceramics can have excellent in vitro apatite-forming ability, which greatly facilitates excellent bioactivity and bone regeneration ability when used as bone scaffolds.

### 3.11. Utility of the Present Approach

The lithography-based ceramic 3D printing technique with a specifically designed top-down process used in this study can produce porous CaP scaffolds with tailored pore orientations, which can have tailored mechanical properties (e.g., compressive strength and modulus). Compared to extrusion-based 3D printing techniques, which can produce porous ceramic scaffolds comprised of continuous filaments, the lithography-based ceramic 3D printing technique can construct more complex external shapes tailored for individual patients [16,28]. In addition, frameworks with a square cross-section instead of a circular cross-section can provide a large bonding area between the layers and thus high mechanical strengths can be achieved.

**Figure 15:**
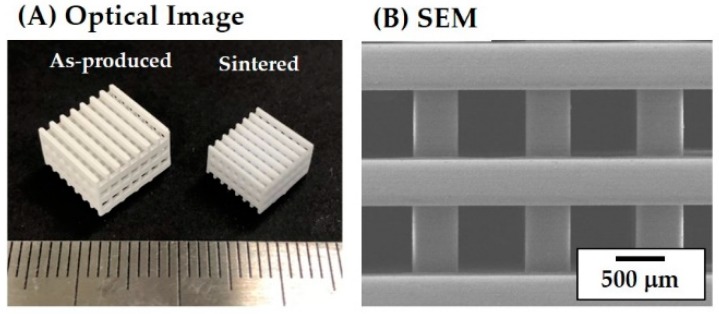
(**A**) Optical image of the as-produced and sintered CaP scaffolds and (**B**) SEM image of the sintered CaP scaffolds (scale in Figure 15A = 1 mm).

Unlike other lithography-based AM techniques, the top-down processing particularly used in this study can directly deposit a thin layer of a ceramic slurry onto a previously photo cured layer. This approach can mitigate the risk of delamination between the photo cured layers due to the high viscosity and sticky nature of the highly loaded ceramic slurry. In addition, the use of decalin as the diluent allows for a high solid loading with a sufficiently low viscosity. Thus, highly densified frameworks after sintering at high temperatures can be construed in a controlled pattern (c.f. Figure 5). In addition, the pore size is readily tunable. For example, porous CaP scaffolds with much smaller pore sizes (667 ± 12 μm) could be produced using our approach (Figure 15A,B), which are advantageous for bone ingrowth into pores [5,6,48].

It should be noted that our approach can be applied to a variety of bioactive ceramics including hydroxyapatite (HA), tricalcium phosphate (TCP), and bioactive glasses. In addition, owing to the great design flexibility of the present approach, the porous structure of porous ceramic scaffolds can be tailored and optimized, which provides excellent mechanical properties while preserving their 3-dimensionally interconnected pore network for fast bone regeneration. Thus, the proposed lithography-based ceramic 3D printing technique with a specifically designed top-down process can find very useful applications in bone tissue engineering.

## 4. Conclusions

Porous CaP ceramic scaffolds with tailored pore orientations were successfully produced using our custom-built 3D printer that can deposit thin, uniform layers of a highly concentrated CaP slurry on the previously photo cured layers. The use of HDDA and decalin as the photo curable monomer and diluent, respectively, enabled the achievement of the high solid loading (*φ* = 45 vol%) and suitable rheological behavior (i.e., shear thinning). In order to precisely control the porous structure of the porous CaP scaffolds, several processing parameters were carefully controlled including the thickness of the CaP slurry layer in the layer-by-layer printing process (200 μm), exposure time (7 s), and the initial width of the CaP framework (450 μm). Three different pore orientations (i.e., 0°/90°, 0°/45°/90°/135° and 0°/30°/60°/90°/120°/150°) were successfully fabricated while consistent porosities (~ 70 vol%) and CaP frameworks and channel dimensions (~484 (±8) μm × 583 (±12) μm and ~951 (±9) μm × 1194 (±18) μm, respectively) were achieved. The compressive strength and modulus of the porous CaP scaffolds were strongly affected by their pore orientation. In other words, the CaP frameworks with an orientation of 0° can more effectively withstand applied compressive loads and, thus, the porous CaP scaffold with a pore orientation of 0°/90° exhibited the highest compressive strength (14.9 ± 1.61 MPa) and modulus (274 ± 24.4 MPa). On the other hand, multiple pore orientations could provide more isotropic mechanical properties regardless of the loading directions. In addition, the porous CaP scaffolds demonstrated an excellent in vitro apatite-forming ability. Taken together, it can be concluded that porous CaP scaffolds with tailored pore orientations can be effectively produced using the proposed lithography-based ceramic 3D printing process, which provides tailored mechanical properties with good bone regeneration ability when used as bone scaffolds.

## Figures and Tables

**Table 1:** The dimensions of the CaP filaments and channels in the x–z direction and the overall porosity of the designed scaffold, as-built scaffolds and produced scaffolds after sintering at 1250 °C for 3 h.

Porous Structure	Initial Design	As-Built Scaffold	Produced Scaffold
Dimension of CaP Frameworks [μm] ^§^	450 × 1000	583 (±12) ×1059 (±18)	484 (±8) × 583 (±12)
Dimension of Channels [μm] ^§^	1500 × 1000	1194 (±17) ×1059 (±18)	951 (±9) ×1194 (±18)
Overall Porosity [vol%]	~75	80 ± 18	70 ± 2.1

§ Dimensions in the x–z direction.

**Table 2:** Schedule for the de-binding process for the complete removal of the photo cured polymers and additives in the green scaffolds.

Step	Heating Rate [°C /min]	Temperature [°C]	Dwelling Time [min]
1	5	335	60
2	1	415	120
3	2	600	60
4	5	1250	180

**Table 3:** Compressive strengths and modulus of the porous CaP scaffolds with different pore orientations of 0°/90°, 0°/45°/90°/135°, and 0°/30°/60°/90°/120°/150°.

Pore Orientation	0°/90°	0°/45°/90°/135°	0°/30°/60°/90°/120°/150°
Compressive Strength [MPa]	14.9 ± 1.61	6.2 ± 1.10	4.8 ± 0.35
Compressive Modulus [MPa]	274 ± 24.4	204 ± 28.4	179 ± 19.9
